# Toward a necessary right to self-representation in European civil law

**DOI:** 10.3389/fsoc.2026.1742364

**Published:** 2026-05-18

**Authors:** Sebastián Rivero Silva

**Affiliations:** Universidad San Jorge, Villanueva del Gállego, Spain

**Keywords:** access to justice, litigants in person, *pro se* litigants, self-representation, Standby Counsel

## Abstract

This article argues that the highly restrictive approach to self-representation in civil proceedings within European civil law systems lacks sufficient historical, dogmatic, and functional justification, being increasingly incompatible with contemporary standards on access to justice. A historical-comparative reconstruction of the Romano-canonical model and the evolution of legal professions shows that there was no original veto on litigants choosing to conduct their own case. In other words, current mandatory-representation rules largely stem from guild-like dynamics of monopolizing audience rights. A contrast with common law systems—where “pure” forms of self-representation coexist with mechanisms such as the McKenzie friend (UK) and Standby/Advisory Counsel (US) —highlights the particular rigidity of the continental model. Paradoxically, civil law systems are characterized by strong judicial management, codified frameworks and the principle of iura novit curia, which make it easier to correct lay errors without excluding self-represented parties from the forum. On this basis, the article advances a twofold normative thesis: (i) in light of Article 6 ECHR and contemporary access-to-justice standards, a limited but effective right to self-representation in less complex civil disputes is possible; and (ii) this right should be structured through assisted self-representation schemes, particularly via an adapted reception of the Standby Counsel model, capable of balancing party autonomy, equality of arms, and procedural efficiency. The proposal requires rethinking the role of intermediate legal professions, subjecting corporate monopolies to scrutiny. Also, recognizing that the democratic legitimacy of civil justice demands meaningful space for the citizen's direct voice before the courts.

## 1 Introduction: framing the problem

The fact that Article 4(1) of the Principles of Transnational Civil Procedure of American Law Institute UNIDROIT (2004) frames legal representation as a “right” fully at the disposal of the party, while Article 14 of the Model European Rules of Civil Procedure (European Law Institute UNIDROIT, 2020) considers it necessary to add the caveat “unless required by law”, is anything but coincidental. As Kilian (2016) notes, European civil law jurisdictions derived from Roman law are generally inclined to restrict self-representation in civil cases—still more so in criminal proceedings. With notable exceptions such as small claims, they oppose the approach of common law, which tend to be more receptive to appearances *pro se* (in US doctrine) or as litigants in person (in UK doctrine). In US federal civil jurisdiction, according to Hammond (2021), at least 25% of civil cases proceed on a *pro se* basis, without legal representation. In most federal districts there are no unified, specific rules applicable only to such litigants; in principle, they are subject to the same procedural requirements as represented parties.

Assy (2011) points out that, not without media controversy, Schedule 3 of the Legal Services Act 2007 permits civil litigants in England and Wales to appear on their own behalf. This understanding has been confirmed by British case law, notably in *Barton v Wright Hassall LLP* [2018] UKSC 12, where the Supreme Court of the United Kingdom held that a litigant in person is “fully responsible” for the conduct of his case if he chooses to act without a lawyer.^1^ Likewise, in *R (Pelling) v Bow County Court* [1999] EWCA Civ 879, the Court of Appeal (Civil Division) recognized the right to self-representation but held it non-transferable: only the party may represent him- or herself; representation cannot be delegated to an unqualified third party. However, *McKenzie v McKenzie* [1970] 3 WLR 472 (CA) established the concept of the “McKenzie friend” (UK Judiciary, 2010): generally a lay person who may assist a self-represented party emotionally, by taking notes, or by offering discreet advice, but who may not address the court on that party's behalf.

In the United States, the pattern is similar. Under 28 U.S.C. § 1654, parties have a right to conduct their own cases, subject to limits such as the exclusion of this right for certain entities. In *C.E. Pope Equity Trust v United States*, 818 F.2d 696 (9th Cir. 1987), the US Court of Appeals for the Ninth Circuit held that the statutory right is strictly personal and does not extend to artificial entities. For corporations and other juridical persons, *Rowland v California Men's Colony*, 506 U.S. 194 (1993), confirmed that they must appear through licensed counsel; similarly, *Eagle Associates v Bank of Montreal*, 926 F.2d 1305 (2d Cir. 1991), supports the conclusion that partnerships and other entities cannot rely on *pro se* representation. As in the UK, US courts have further clarified that the right of self-representation cannot be delegated to a “non-lawyer”, as confirmed in *Simon v Hartford Life, Inc*., 546 F.3d 661 (9th Cir. 2008).

What constitutes the general rule in the UK and the US—the possibility of self-representation—remains the exception in continental European civil law. Typically, only minor monetary claims, pre-litigation conciliation procedures, and low-relevance proceedings without substantive court appearance permit parties to act without specialized representatives (Ginzburg, 2024). A clear illustration is provided by two EU instruments. First, the European Order for Payment Procedure governed by Regulation (EC) No 1896/2006, which allows cross-border monetary claims to be pursued without mandatory legal representation, regardless of the value of the claim. Secondly, the European Small Claims Procedure established by Regulation (EC) No 861/2007 (as amended), which applies to cross-border claims up to EUR 5,000 and likewise does not, in principle, require professional representation.

Taken together, these instruments indicate that, at least for low-value and standardized disputes, EU procedural policy assumes that self-representation is both feasible and compatible with efficient adjudication. The restriction is especially strict in Italy (approx. EUR 1,100), Spain (approx. EUR 2,000), and Portugal (approx. EUR 5,000).^2^ This is not a trivial point, scholars such as McKeever et al. (2022), drawing inter alia on Article 6(3)(c) ECHR in the criminal context and on the broader guarantees of Article 6 ECHR, argue that the right “to defend himself in person or through legal assistance of his own choosing” underscores the centrality of effective participation for the fairness of proceedings. By analogy, where a civil litigant wishes to appear in person, the categorical exclusion of that possibility requires careful justification.

Assy (2015) argues that this European stance, unmistakably paternalistic, seeks to protect the *pro se* litigant who “does not know what he is getting into” and cannot grasp the consequences of his choice. Once proceedings exceed straightforward “one-shot” disputes such as simple debt claims, ignorance of procedural and substantive law may (a) undermine equality of arms and (b) cause serious procedural inefficiencies, with repeated adjournments whenever the *pro se* litigant speaks out of turn, misapplies the rules, or claims non-existent entitlements. Bradlow (1988) suggests that judges facing *pro se* litigants generally oscillate between two models. Under a “strict” approach, the judge lets the *pro se* party “crash” within a system designed for highly trained lawyers. Under a “flexible” approach, the judge intervenes to rebalance an unequal contest between expert counsel and an untrained layperson. The latter, however, may itself strain the principle of equality between the parties as formulated by Chiovenda (1923). Against this background, the present article asks: to what extent is it legally possible and normatively desirable that European civil law systems recognize a broader right to self-representation in civil proceedings?

The ECtHR has accepted that mandatory legal representation before courts of cassation or supreme courts is compatible with Article 6 ECHR, provided there are effective legal aid mechanisms rendering such a requirement realistic (see *Tabor v Poland*, App. No. 12825/02, 27 June 2006). By contrast, before the EU Courts, Article 19 of the Statute of the Court of Justice of the European Union, as interpreted inter alia in *Uniwersytet Wrocławski and Poland v REA* (C-515/17 P and C-561/17 P), *Universität Bremen v REA* (C-110/21 P) and *Studio Legale Ughi e Nunziante v EUIPO* (C-776/22 P), almost completely excludes self-representation for non-privileged parties, entrenching a model openly hostile to *pro se* litigation even for institutional or professional actors. Recent scholarship (McKeever et al., 2022) advocates abandoning paternalistic stances and facilitating self-representation where possible, reading Article 6 ECHR's guarantee of access to courts as compatible with, and in some respects supportive of, a right to appear in person. On this view, “access to justice does not require every litigant to be represented”, which is taken to imply a qualified right to self-representation.

From the standpoint of Roman law foundations, there is no “original prohibition” that could justify an almost abusively restrictive stance toward those who wish to represent themselves in relatively simple matters, with no, limited, or greater support from a lawyer. Other Roman law scholars reach similar conclusions. Rodriguez Diez (2017) states that “there is no evidence to claim the existence of a primitive ban on direct representation in Roman law”. Chroust (1954) shows that although Roman law gradually developed increasing professionalization and restrictive regimes for legal services, it did not impose an absolute obligation to be assisted by counsel to appear before the authorities, whatever the practical risks of choosing otherwise. Accordingly, the so-called “Roman civil law tradition” cannot, by itself, sustain a structural prohibition of self-representation in Europe. This paternalistic approach to mandatory representation mirrors a fundamental theoretical conflict in comparative law: the ongoing tension between individual autonomy and the State's duty of protection (Guerrero, 2024b).

## 2 The historical acceptance and development of self-representation

In classical Rome, procedural functions—whether for defense or accusation—were structured around three roles: (a) the capacity to speak in public and persuade judge or jury, exercised by the *orator* or *advocatus* (Ulpian, *Dig*. 3.1.1.4); (b) the capacity to know and argue the law, associated with the *iurisprudens* (Papinian, *Dig*. 1.1.7.1); and (c) the existence of a proper mandate from the client, entrusted to the *procurator* or *cognitor* (Gaius, *Inst*. 4.82–85). The client—the party wishing to litigate—had to consent to the chosen representative and accept the outcome flowing from the representative's acts in accordance with rules on appointment and ratification (Paulus, *Dig*. 3.3.2 pr.; Gaius, *Inst*. 4.97–98).

These functions could be combined in one or more persons; there was no requirement that three distinct individuals perform them. Ulpian noted that the procurator appears when the prospective litigant “does not wish or is not able” to attend to his affairs personally (*Dig*. 3.3.1.3). Thus, early codified law does not, *prima facie*, justify restrictions in favor of any professional group against the litigant himself, nor does it mandate forcing him to hire representation if he does not wish to do so. Quintilian nevertheless urged that professional defense is preferable to relying solely on self-representation, arguing that “the laws themselves would be powerless without the aid of advocates capable of upholding them” (*Institutio Oratoria* 12.3.2–3, trans. Butler, 1920/2004). Frier (1989) similarly emphasizes the increasing expertise and professionalization of Roman jurists, logically rendering the system less hospitable to purely archaic *pro se* litigation.

After the fall of Rome, both medieval European and English law broadly preserved the original distinction between *procurator* (trusted agent) and *advocatus* (pleader and jurist). In the Anglo-Saxon context, Chroust (1956) suggests that, under the influence of ecclesiastical courts shaped by Roman-canonical tradition, medieval England developed an early distinction between advisor-advocate or pleader (a precursor of the modern solicitor and of continental advocates) and the *attornus* (a precursor of the American attorney-at-law and the English barrister), initially conceived as a simple agent for specific court appearances, whose role gradually expanded and professionalized.

In medieval continental Europe, the *ius commune*—again heavily influenced by canon law—maintained the distinction between the legal advisor (*advocatus* or *bozero*) and the representative handling minor procedural acts, the *procurator* (Llamosas, 2018). The *advocatus* not only advised on the law but also addressed and persuaded the court; the *procurator* was a mere agent to “see and hear” on behalf of an absent party in civil matters, whilst personal appearance remained obligatory in criminal proceedings. Brundage (2019) locates in this medieval context—both continental and English—the origins of the legal profession as we understand it today. This is not a marginal observation. Medieval canon law—particularly the collections later known as the *Corpus iuris canonici* (the *Decretum Gratiani, Liber Extra* of Gregory IX, *Liber Sextus* of Boniface VIII, *Clementinae, Extravagantes*)—drew a clear line between the *procuratores ad lites* (proctors) and the *advocati*. In ecclesiastical courts, proctors acted as procedural agents under express mandate, while *advocati* were trained jurists subject to specific ethical obligations, who could also act as proctors. The converse was not true: proctors, lacking the required qualifications, were barred from acting as *advocati* (Pound, 1943).

This point is crucial. France, Germany, Spain and other civil law jurisdictions adopted this canonical asymmetry between the legal advisor and the mere representative for procedural formalities. The UK, by contrast, progressively broke away from this model. Pound (1944) shows that, in the sixteenth and seventeenth centuries, consolidation of the Inns of Court's control over admission to the bar, and differentiation between barristers, attorneys and solicitors linked to the growth of the Court of Chancery, cemented a clear division between the professions that anticipate the modern barrister and solicitor. In simplified terms, this marks (a) the European model: a lawyer with full rights of audience, assisted by a procedural auxiliary (*procurador*) holding a ritualized mandate of representation; and (b) the British model: a split between litigation specialists (barristers) and legal advisers (solicitors), the latter retaining audience rights in the lower courts. The later US model would unify these roles in the attorney-at-law.

Following Pound, Chroust (1959) maintains that it is precisely between the fifteenth and seventeenth centuries that solicitors (legal experts) and barristers (those entitled to address the court) consolidate their professional domains and secure a monopoly over courtroom advocacy in England. Today, the framework is governed principally by the Legal Services Act 2007, which designates “the exercise of a right of audience” as a reserved legal activity. As Ching (2021) explains, solicitors, once admitted by the Solicitors Regulation Authority (SRA), acquire automatic audience rights in the lower courts but require additional accreditation under the Courts and Legal Services Act 1990, s. 67, to appear in the higher courts. Barristers, regulated by the Bar Standards Board (BSB), hold full rights of audience upon being called to the Bar and obtaining a practicing certificate; they do not require a solicitor to “front” them, unlike continental *procuradores*. Furthermore, alternative regulatory routes—such as CILEX (Bones, 2022)—contribute to a more dynamic market with less resistance to professional monopolies.

Civil law jurisdictions have constructed the role of the representative (the original Roman *procurator*) in a way that sharply diverges from the Anglo-Saxon barrister. In Spain and Andorra, for example, the *procurador* formally “represents” the party, yet in practice performs auxiliary functions for the *abogado* or *advocat*. In both countries, *procuradores* are barred from speaking before the judge: only lawyers hold that right of audience. When *procuradores* act without the lawyer present, they do so under the lawyer's instructions, to lodge documents or receive notifications—merely to “see and hear”—as provided in Articles 23 and 31 of the Spanish Civil Procedure Act and Articles 93–101 of the Andorran *Llei Qualificada de la Just*í*cia*. Their “representation” is thus ritual rather than substantive. In Spain, *procuradores* frequently never even meet the clients they formally represent; they are chosen directly by lawyers for their procedural efficiency.

In this context, Spanish Supreme Court Judgment 402/2016 confirms that the absence of the *procurador* from the hearing does not entail lack of representation where counsel is present. In any event, in light of the foregoing, it is my view as the author that there are fundamental distinctions between the functions and nature of a barrister and a *procurador*, which preclude drawing any kind of equivalence. The *procurador* has no direct equivalent in the common law world, because representation and audience rights are integrated within the legal profession (solicitor in lower courts, barrister in all courts). The *procurador* neither pleads, nor advises, nor drafts substantive submissions; he cannot be assimilated to either barrister or solicitor.

At first sight, therefore, one might expect the British barrister or US attorney—whose monopoly is directly affected—to be more hostile to self-representation in civil matters. By contrast, the ritualized role of the Spanish or Andorran *procurador*—with all due respect to the value of their procedural assistance—appears far more replaceable by the litigant, perhaps with varying degrees of support from counsel, who retains exclusive rights of audience. The structural division in civil law systems between a procedural assistant who “represents” without speaking, and the professional who exercises *public appearance*, makes it intuitively easier to envisage pathways toward assisted self-representation.

Recent socio-legal scholarship confirms that the historical consolidation of professional monopolies remains a central friction point for modern access to justice in Continental Europe. Current doctrinal debates highlight that contemporary civil justice systems are under significant strain due to escalating procedural costs and structural inefficiencies (Ahmed et al., 2025). Within this context, researchers emphasize that the traditional requirement of professional legal assistance constitutes a major financial burden, frequently acting as a structural barrier to court access rather than a facilitator (Biard et al., 2021). To address these financial hurdles, recent European procedural innovations—such as the European Small Claims Procedure—actively bypass national mandatory representation rules to empower litigants and reduce expenses (Kramer and Dori, 2025). Ultimately, this suggests that civil justice frameworks must evolve to accommodate and assist citizens directly without exclusively relying on mandatory intermediation.

## 3 The procedural impact of self-representation

Anglo-American scholarship, despite recognizing a relatively strong right to self-representation, is not oblivious to European-style paternalist concerns. Konnerth (2019), for instance, criticizes mandatory mediation in parts of the New York State Court System on the ground that it may push unrepresented parties into settlements inconsistent with their legal interests. Quintanilla (2019) suggests that the high incidence of *pro se* litigants in US courts is a systemic dysfunction and should not be accepted uncritically. According to this view, judges and court staff may develop negative stereotypes toward self-represented parties, resulting in poorer treatment and diminished judicial empathy. Kroeper et al. (2020), in an experimental study involving judges and lawyers from Indiana, confirm the existence of biases against *pro se* litigants. Kuehn (2006) adds that the *pro se* phenomenon is linked, among other factors, to a shortage of trial lawyers which inflates the cost of legal services. As will be seen, economic constraints are central but do not fully explain the phenomenon.

Buhai (2009) squarely addresses the core issue underlying the right to self-representation: whether judges must actively assist or protect unrepresented parties to secure a fair trial. In an adversarial model bordering on arbitral logic, the unrepresented litigant has virtually no chance in complex matters when facing experienced counsel. By contrast, Buhai argues that the civil law model—with its more active judiciary, constrained by statute and often labeled “inquisitorial”—is structurally better placed to ensure a degree of balance between represented and unrepresented parties. European procedural law, more rigidly codified, compels judges to structure the dispute and manage the proceedings *ex officio* (Verkerk and van Rhee, 2012). Where an unrepresented party is substantively correct, it is correspondingly harder for a civil law judge to decide the case in open contradiction to the applicable law solely due to minor procedural missteps. Additionally, many European jurisdictions adhere to *iura novit curia* (García, 2023), binding the judge to apply the law irrespective of parties' legal submissions. The active management role of the civil law judge is part of a broader continental tradition of active judicial control. European jurisdictions have historically developed a strong *iurisdictio* capable of deploying innovative procedural tools and exercising effective oversight, even in highly complex scenarios (Guerrero, 2024a).

Swank (2004) suggests that *pro se* litigation in the US is predominantly driven by economic constraints and concentrated in “poor people's courts” dealing with minor matters (traffic, family, etc.). A second driver lies in ideological or political motivations. In any event, *pro se* practice is widely associated with procedural problems: missed deadlines, defective filings, and delays. Goldschmidt (2011), addressing the “gray area *pro se*” following *Indiana v Edwards*, 554 U.S. 164 (2008), notes that cases combining incompetence with emotional or mental health problems prompted courts to impose some form of compelled assistance, thereby curbing unfettered self-representation to avoid “spectacle” in the courtroom. This evolution reshapes the famous *Faretta* rule derived from *Faretta v California*, 422 U.S. 806 (1975): the right to self-representation does not confer “a license not to comply with relevant rules of procedural and substantive law”. Partial assistance and reasonable accommodations that do not undermine equality of arms emerge as plausible solutions to (a) preserving the right to self-representation in common law jurisdictions and (b) introducing it, under safeguards, into civil law systems.

In Europe, self-representation is also closely tied to economic considerations. Legal aid schemes often apply income thresholds that exclude large segments of the population: many can afford a lawyer and *procurador* only at serious sacrifice, and regard mandatory representation in minor “one-shot” disputes—and the separate obligation to pay a *procurador* for tasks they could perform themselves—as unjust. Toy-Cronin (2016b) warns that although economic factors are significant, other legitimate motives include past negative experiences with lawyers, the belief that one's case is simple enough to handle personally, or—here I add—a principled exercise of a legally recognized choice, which should not attract criticism if undertaken knowingly. Toy-Cronin (2022), drawing on interviews with judges and lawyers in New Zealand (a common law jurisdiction), identifies three heuristic categories of litigants in person (LiPs): (a) vexatious or erratic litigants; (b) those in genuine financial need; and (c) the “lawyer-like”, who prepare their cases seriously and acquire sufficient procedural knowledge to enable hearings with minimal disruption.

Cooper (2014) suggests that most *pro se* litigation revolves around minor disputes and that minimal legal assistance (even from law students) would significantly improve efficiency, alleviating delays caused by inexperience. Brimo (2022) introduces the prospect of AI assistance for unrepresented parties and underscores the need to address ethical issues: the opacity of algorithmic “black boxes” and the unknown composition of training datasets make AI an unreliable ally for those exercising a right to self-representation. Hurder (1998) even contemplates reconfiguring the McKenzie friend model, exploring the risks and opportunities of assistance by non-lawyers, as informally seen in New York City Housing Court. Yet both non-expert assistance and AI tools encounter a major obstacle in civil law jurisdictions: the strict concept of reserved activities, which often includes not only rights of audience but virtually all forms of legal advice.

Willis (2023) argues that the “right to be heard”, from which self-representation flows, is “at its core, a moral right and a human right which is commonly equated to the right to a fair trial”. The European framework has been cautious in fully embracing this implication. The Handbook on European law relating to access to justice (European Union Agency for Fundamental Rights, Council of Europe, and European Court of Human Rights, 2016) underscores that parties may appear in person or be represented, and that any restrictions—such as mandatory representation—must be justified by the interests of justice and comply with proportionality. Read together with Article 6 ECHR and Article 47 CFR, this suggests that qualified forms of self-representation are not only permissible but may, in certain contexts, be normatively supported rather than discouraged. At present, the Council of Europe (2025) has taken a very tentative position on the issue, recommending minor improvements to provide litigants in person with specific facilities. For example, as the judge ‘explaining the conflict in plain language' or the availability of simplified hard-copy forms for communicating with the judicial body. In any event, under no circumstances has a solid, pioneering approach been proposed concerning a prospective development of the European right to self-representation.

## 4 Standby counsel in common law and its transposition to civil law

Toy-Cronin (2019) describes a dynamic which, though observed in New Zealand, is generalisable to other LiPs and *pro se* litigants: self-represented parties often feel excluded from what appears to be a closed exchange between opposing counsel and judge, which, in turn, can fuel “erratic”, “showman-like”, or “conspiratorial” behavior, as discussed above. To prevent such alienation, Anglo-American doctrine has developed the figure of Standby Counsel (Goldschmidt, 2014): a lawyer appointed—sometimes compulsorily—by the court to assist, to varying degrees, a self-represented litigant so as to reduce procedural disadvantage without displacing that litigant's control of the case. In *McKaskle v Wiggins*, 465 U.S. 168 (1984), the US Supreme Court articulated key limits of Standby Counsel: (a) counsel must not override the defendant's control or usurp the visibility of self-representation before the jury; (b) limited unsolicited interventions are permissible; and (c) technical support should generally occur outside the formal hearing. Building on Mayberry v Pennsylvania, 400 U.S. 455 (1971), Goldschmidt (2014) argues that, subject to the *lex fori*, courts may in exceptional circumstances impose standby counsel against the wishes of a self-represented litigant in order to safeguard the integrity and decorum of proceedings. This is best understood as a doctrinal inference from the Court's insistence on orderly process, rather than as an unqualified, free-standing rule.

Joy and McMunigal (2021) explores the ethical duties of lawyers acting as Standby Counsel. New York State Bar Association Opinion No. 949 distinguishes three roles: (a) passive standby, available if requested; (b) intermediate, providing targeted assistance (e.g. on technical points or drafting complex filings); and (c) active, where standby counsel effectively manages the case and must meet the ordinary ethical standards of full representation. According to ABA Formal Opinion 07-448, an attorney–client relationship does not arise automatically from appointment as Standby Counsel; it crystallizes when the litigant accepts or is required to accept the assistance offered. Eshbach (2017), examining Pennsylvania practice, highlights the problems generated by recognizing Standby Counsel without clear regulation, as in Rule 121(D) of the Pennsylvania Rules of Criminal Procedure, which merely states that standby counsel “shall attend the proceedings and shall be available to the defendant for consultation and advice”. Such vagueness leaves judges to define the contours of the role, subject to the boundary drawn in *United States v Tilley*, 326 F. App'x 96 (3d Cir. 2009): (a) Standby Counsel may not act contrary to the *pro se* litigant's wishes; (b) the *pro se* litigant cannot complain of having been assigned Standby Counsel when he chose to represent himself.

Howard (2001) analyses the confusion in South Carolina between hybrid representation and Standby Counsel. Article I, §14 of the state constitution grants an accused the right “to be fully heard in his defense by himself or by his counsel, or both”. Decisions such as *State v Sanders* (1977) and *State v Reed* (1998), where standby counsel was authorized to “aid [the] appellant to the extent he desired”, blurred the line, encouraging quasi-hybrid models or ill-defined standby roles. Howard argues against recognizing a right to hybrid representation and calls instead for a clearer definition of standby—relabelled advisory—counsel. In the same vein, Lisowski (2013), discussing *Cole v Patricia A. Meyer* and *Associates*, 206 Cal. App. 4th 1095 (2012), warns of the dangers when a lawyer acts as Standby Co-Counsel alongside another lawyer—or a lay litigant—thus entangling professional responsibility with possibly unwise litigation strategies.

The prevailing prohibition of self-representation in continental Europe (Luban, 2018) has, as a corollary, the rejection of Standby Counsel. Yet European systems could also benefit from this figure, properly adapted. As argued above, the civil law separation between *procurador* (procedural assistant and formal representative) and lawyer (advocate and strategist) actually facilitates a move toward assisted self-representation. One can readily imagine a citizen personally filing claims, enquiring about the status of his case, or requesting copies of the court file while receiving targeted legal support. If the main objection to self-representation in Europe is that it would cause delays and disrupt equality of arms, why not target those specific risks? Poulin (2000) conceptualizes Standby Counsel as the litigant's “shadow”: in my own terms, the ideal mechanism to reconcile (a) an undeniable “natural” right to self-representation, currently curtailed in civil law systems, with (b) an interesting mitigation of the negative externalities associated with its exercise.

Farber (2020) likewise advocates for introducing Standby Counsel in support of self-representation rights. Rondinelli (2019) argues against the demonisation of hybrid models, presenting them instead as “a potential workable solution” for effective use of self-representation. In this sense, the Civil Justice Council (2011) stresses that enabling parties to take an active role and address the court directly is both (a) a right and (b) a means to enhance trust and transparency in the justice system by allowing citizens to “cross the threshold” and act in their own interest. Recent qualitative research by Janssen et al. (2025) on self-represented litigants in early stages of Dutch small claims proceedings shows that perceptions of procedural justice—being treated with respect, having a real opportunity to explain one's position, and receiving clear information—are rlevant for trust in judges. Their findings suggest that hearing designs which ensure fairness and clarity can sustain, or even strengthen, the legitimacy of civil justice in a context of increasing self-representation, rather than undermining it.

European reluctance to embrace self-representation can, in part, be understood through two structurally reinforcing dynamics. First, the political and regulatory influence of organized legal professions favors the preservation of reserved activities and segmented roles, which may contribute to de facto monopolistic structures under the banner of quality assurance (Garoupa, 2004). Secondly, institutional convenience: courts and court staff are accustomed to interacting with legally trained intermediaries and often perceive self-represented parties as administratively burdensome or “problematic” (Moorhead and Sefton, 2005; McKeever et al., 2018). These professional and institutional preferences shape the regulatory environment, even if they are rarely articulated as such in the formal justifications for mandatory representation. Toy-Cronin (2016a) even notes that something as simple as where a self-represented litigant is seated in the courtroom can become contentious—though, in my view, such logistical difficulties cannot justify withholding the right itself in civil law systems.

## 5 Discussion and conclusion

The historical-comparative reconstruction supports an affirmative answer to the research question: regulated recognition of self-representation in civil law jurisdictions is not only normatively defensible but also recommendable in light of its development in common law systems. Analysis of Romano-canonical tradition and the medieval and modern evolution of legal professions shows that the highly restrictive approach to *pro se* litigation in European civil law does not arise from any inherent logic of fair process. Rather, it is the product of progressive corporatisation and monopolization of audience rights. Neither the classical *orator*–*iurisprudens*–*procurator* scheme nor early Roman or canonical codifications imposed an original veto on litigants wishing to conduct their own cases. The contemporary ban may be attributable to a late crystallization rooted in professionalization, guild interests, and monopolies over the interpretation and management of legal conflict.

When this genealogy is confronted with the contemporary concept of access to justice—which includes an effective right to be heard, not merely to speak through intermediaries—the conclusion is clear. A model that makes mandatory professional representation the default rule even in low-value or low-complexity disputes is acceptable only if it passes a strict proportionality test. In dogmatic terms, the financial and symbolic burden imposed on citizens simply to enter the civil forum is, in many instances, difficult to reconcile with a deep reading of Article 6 ECHR and with the imperative to prevent justice from becoming effectively privatized by certain professional bodies. By way of example, in Spain, in a wide range of ordinary civil proceedings mandatory representation by both lawyer and procurador remains required, so that routine procedural steps which could in principle be performed by the litigant—including the lodging of documents or practical management of case information—are structurally mediated through professionals. This does not mean that direct access is legally impossible, but it illustrates how institutional design presumes intermediation even where the underlying activities are neither conceptually nor technically beyond the reach of lay parties.

Functional comparison with common law systems does not vindicate the continental closure; it exposes its inconsistency. In adversarial environments heavily dependent on technical expertise and dense jurisprudence, a strong right to self-representation is recognized and managed through instruments such as the litigant in person, the McKenzie friend, and Standby/Advisory Counsel. Paradoxically, civil law systems—with stronger judicial management, codified frameworks, and *iura novit curia*—refuse to allow litigants to assume responsible control of their cases even where risks can be rationally more contained. The paternalist objection—that self-representation undermines equality of arms and generates procedural chaos—loses much of its force when we acknowledge that civil law procedure already provides tools to correct lay errors, sanction abuse, filter vexatious litigation, and secure basic technical quality without suppressing the very faculty of appearing in person. Maintaining a structural prohibition, instead of designing targeted restrictions for genuinely complex matters (e.g., certain appeals, specialized litigation, or situations of heightened vulnerability), instrumentalises real management problems—and the discomfort of court professionals accustomed to dealing exclusively with lawyers—to deny a right grounded in personal autonomy and democratic legitimacy: the individual as protagonist of his or her own conflict before the judiciary.

Within this framework, the Standby Counsel emerges as the conceptual keystone for a reform consistent with civil law structures. Properly adapted, it is not a charitable indulgence nor a covert restoration of mandatory representation, but a structural device reconciling autonomy and safeguards: the litigant preserves control and direct voice, while a regulated professional—subject to ethical and disciplinary duties—prevents the process from devolving into inefficient improvisation or undignified spectacle. The peculiar continental split between lawyer and (in some countries) the *procurador*, with “representation” ritualized in the latter and monopoly of the *ius loquendi* in the former, reveals precisely the room to recalibrate functions. Partially displacing purely ceremonial intermediaries in favor of assisted self-representation—supported by Standby Counsel empowered to intervene technically without dispossessing the citizen's voice—would reduce costs, clarify the true function of legal professions, and neutralize fears that opening space for self-representation would collapse the system. In contrast to unregulated non-professional assistants or opaque AI-based tools—which strain reserved-activity regimes without offering solid guarantees—controlled hybrid models anchored in professional responsibility represent a normatively superior option.

The resulting conclusion is unequivocal. In light of the reconstructed tradition, the comparative evidence, and European fundamental rights standards, civil law jurisdictions can no longer plausibly justify a highly restrictive approach to self-representation in civil proceedings as the default rule. It is both legally possible and normatively desirable to recognize a limited but genuine right to self-representation, particularly in less complex cases, and to structure it through mechanisms such as Standby Counsel that function as instruments of equality of arms and procedural efficiency, not as disguised forms of compulsory representation. This, in turn, demands critical reassessment of legal professions, closer scrutiny of bar monopolies that condition access to judicial protection, and acceptance that the legitimacy of civil justice—in terms of trust, transparency, and perceived procedural fairness—requires opening the courtroom to the direct voice of the litigant. A civil process that structurally excludes citizens from representing themselves, even where they are willing to assume informed risks and institutional safeguards exist to mitigate them, cannot plausibly be regarded as a neutral technical arrangement. It becomes a form of heteronomous guardianship that sits uneasily with a constitutional culture of adult citizenship.

## 6 Limitations

One limitation of the present analysis should nevertheless be acknowledged. Although it discusses Spain, France, Italy, and Portugal, it does not provide systematic statistical evidence on the incidence of self-representation in those jurisdictions. Its aim is primarily doctrinal and comparative, focusing on the normative structure of representation rules rather than on measuring litigation patterns. Future research should therefore complement this analysis with country-specific empirical data on the prevalence and procedural experience of unrepresented litigants.

## Caselaw

C.E. Pope Equity Trust v. United States, 818 F.2d 696 (9th Cir. 1987). Retrieved from: https://law.resource.org/pub/us/case/reporter/F2/818/818.F2d.696.86-4122.86-4116.86-3552.html. Last access: 08/11/2025.

Cole v. Patricia A. Meyer and Associates, APC, 206 Cal. App. 4th 1095 (Cal. Ct. App. 2012). Retrieved from: https://case-law.vlex.com/vid/cole-v-patricia-meyer-894302456 Last access: 08/11/2025.

Eagle Associates v. Bank of Montreal, 926 F.2d 1305 (2d Cir. 1991). Retrieved from: https://law.justia.com/cases/federal/appellate-courts/F2/926/1305/258403/. Last access: 08/11/2025.

Faretta v. California, 422 U.S. 806 (1975). Retrieved from: https://supreme.justia.com/cases/federal/us/422/806/. Last access: 08/11/2025.

Indiana v. Edwards, 554 U.S. 164 (2008). Retrieved from: https://supreme.justia.com/cases/federal/us/554/164/. Last access: 08/11/2025.

Mayberry v. Pennsylvania, 400 U.S. 455 (1971). Retrieved from: https://supreme.justia.com/cases/federal/us/400/455/. Last access: 08/11/2025.

McKaskle v. Wiggins, 465 U.S. 168 (1984). Retrieved from: https://supreme.justia.com/cases/federal/us/465/168/. Last access: 08/11/2025.

Rowland v. California Men's Colony, Unit II Men's Advisory Council, 506 U.S. 194 (1993). Retrieved from: https://supreme.justia.com/cases/federal/us/506/194/. Last access: 08/11/2025.

Simon v. Hartford Life, Inc., 546 F.3d 661 (9th Cir. 2008). Retrieved from: https://case-law.vlex.com/vid/simon-v-hartford-life-895144201. Last access: 08/11/2025.

United States v. Tilley, 326 F. App'x 96 (3d Cir. 2009). Retrieved from: https://www.casemine.com/judgement/us/59146725add7b049342acb70/amp. Last access: 08/11/2025.

Barton v. Wright Hassall LLP [2018] UKSC 12, [2018] 1 W.L.R. 1119. Retrieved from: https://www.supremecourt.uk/cases/uksc-2016-0136. Last access: 08/11/2025.

McKenzie v. McKenzie [1970] 3 W.L.R. 472, [1971] P. 33 (CA). Retrieved from: https://justiceofthepeace.org.nz/modules/judicial%20studies%20module/resources/mckenzie-v-mckenzie.pdf. Last access: 08/11/2025.

R (Pelling) v. Bow County Court [1999] EWCA Civ 1079, [2000] Q.B. 270 (CA). Retrieved from: https://www.casemine.com/judgement/uk/5b46f1ed2c94e0775e7ee343. Last access: 08/11/2025.

European Court of Human Rights. (2006). Tabor v. Poland (App. No. 12825/02). Judgment of 27 June 2006. HUDOC. https://hudoc.echr.coe.int/eng?\protect$\relax=$001-76078.

Court of Justice of the European Union. (2020). *Uniwersytet Wrocławski and Poland v Research Executive Agency (REA)*, Joined Cases C-515/17 P and C-561/17 P, EU:C:2020:73. CURIA/EUR-Lex. https://curia.europa.eu/juris/liste.jsf?language\protect$\relax=$en&num\protect$\relax=$C-515/17%20P.

Court of Justice of the European Union. (2022). *Universität Bremen v European Research Executive Agency (REA)*, Case C-110/21 P, EU:C:2022:555. CURIA/EUR-Lex. Retrieved from: https://curia.europa.eu/jcms/upload/docs/application/pdf/2025-10/bullettin_en_septembre.pdf. Last access: 08/11/2025.

General Court of the European Union. (2022). *Natixis v European Commission*, Case T-449/21, EU:T:2022:394. Order of 20 June 2022. EUR-Lex. https://eur-lex.europa.eu/legal-content/EN/TXT/?uri\protect$\relax=$celex:62021TO0449.

Court of Justice of the European Union. (2025). *Studio Legale Ughi e Nunziante v European Union Intellectual Property Office (EUIPO)*, Case C-776/22 P, EU:C:2025:644. Judgment of 4 September 2025. CURIA / EUR-Lex https://eur-lex.europa.eu/legal-content/ES/TXT/?uri\protect$\relax=$CELEX:62022CJ0776

Tribunal Supremo (Sala Primera). (2016). Sentencia 402/2016, de 15 de junio (RJ 2016/2828). Retrieved from: https://www.boe.es/biblioteca_juridica/comentarios_sentencias_unificacion_doctrina_civil_y_mercantil/abrir_pdf.php?id\protect$\relax=$COM-D-2016-15. Last access: 08/11/2025.

## Classical sources

Gaius. (1988). *The Institutes of Gaius* (W. M. Gordon and O. F. Robinson, Trans.). Duckworth

Justinian. (1877–1895). *Digesta* (T. Mommsen and P. Krueger, Eds.). In *Corpus Iuris Civilis*. Weidmann.

Justinian. (1877–1895). *Institutiones* (P. Krueger, Ed.). In *Corpus Iuris Civilis*. Weidmann

Quintilian. (1920–1922). *Institutio Oratoria* (H. E. Butler, Trans.). Loeb Classical Library.

Corpus Iuris Canonici. (1879–1881). (A. Friedberg, Ed.). 2 vols. Tauchnitz.

## Legislation

European Convention for the Protection of Human Rights and Fundamental Freedoms (Council of Europe).

Charter of Fundamental Rights of the European Union (EU).

Regulation (EC) No 861/2007 of 11 July 2007 establishing a European Small Claims Procedure (EU).

Legal Services Act 2007 (UK).

28 U.S.C. § 1654 (US).

Ley 1/2000, de 7 de enero, de Enjuiciamiento Civil. (Spain).

Llei qualificada de la justícia, del 3 de setembre de 1993 (Andorra).

Pennsylvania Rules of Criminal Procedure, Rule 121. (US – PA).
